# Association of Routinely Measured Proinflammatory Biomarkers With Abnormal MRI Findings in Asphyxiated Neonates Undergoing Therapeutic Hypothermia

**DOI:** 10.3389/fped.2021.624652

**Published:** 2021-03-29

**Authors:** Maria Ruhfus, Stamatios Giannakis, Mona Markus, Anja Stein, Thomas Hoehn, Ursula Felderhoff-Mueser, Hemmen Sabir

**Affiliations:** ^1^Department of Pediatrics I/Neonatology, University Hospital Essen, University Duisburg-Essen, Essen, Germany; ^2^Department of General Pediatrics, Neonatology and Pediatric Cardiology, Medical Faculty, University Children's Hospital Duesseldorf, Heinrich Heine University, Duesseldorf, Germany; ^3^Department of Neonatology and Pediatric Intensive Care, Children's Hospital University of Bonn, Bonn, Germany; ^4^German Centre for Neurodegenerative Diseases (DZNE), Bonn, Germany

**Keywords:** newborn, asphyxia, cooling, outcome, biomarker, aEEG

## Abstract

**Background:** The neuroprotective treatment effect of therapeutic hypothermia (TH) following perinatal asphyxia may be negatively influenced by neonatal sepsis and concomitant inflammation. We aimed to correlate routinely used blood biomarkers for perinatal sepsis in cooled asphyxiated newborns with MRI findings.

**Methods:** Perinatal data was retrospectively collected from 67 cooled asphyxiated newborns. Levels of C-reactive protein (CRP), white blood cells and platelets were analyzed before, during and after TH. Interleukin-6 blood levels were analyzed before initiation of TH. Magnetic resonance imaging (MRI) on postnatal day 5–7 was used defining short-term outcome. Adverse outcome was defined as death or adverse MRI findings. Amplitude-integrated electroencephalography (aEEG) was additionally analyzed and correlated with short-term MRI outcome.

**Results:** Forty-nine newborns had favorable short-term MRI outcome. Perinatal data referring to perinatal sepsis did not differ significantly between groups. IL-6 levels before initiation of TH and CRP levels on day three and after TH were significantly higher in newborns with adverse short-term MRI outcome. Males with adverse short-term MRI outcome had significantly increased CRP values at the end of the cooling phase. aEEG strongly correlated with short-term MRI outcome.

**Conclusion:** Routinely used blood biomarkers may be helpful early identifying newborns at high risk of unfavorable outcome and in need of close neurodevelopmental follow-up.

## Introduction

Therapeutic hypothermia (TH) is the standard treatment for neonatal encephalopathy (NE) following perinatal asphyxia in near-term newborns (1). With a risk reduction of 15%, long-term neurological sequelae (motor and mental disabilities) are significantly reduced in cooled asphyxiated newborns compared to non-cooled asphyxiated newborns (1). However, almost half of all asphyxiated cooled newborns enrolled in the large randomized controlled cooling trials, did not benefit from the treatment (1). Different risk factors have been identified influencing the treatment effect of TH in asphyxiated newborns, such as severity of HIE (2), delayed treatment onset (3), hypo- and hyperglycemia (4), seizures (5), hyperoxia and hypocapnia (6) during TH. However, some of these might just reflect clinical problems within the more severely asphyxiated newborns. Additionally, several co-morbidities might influence the neuroprotective potency of TH. One well-known risk factor for adverse neurological outcome in near-term newborns is perinatal sepsis (7). Experimentally, TH is not neuroprotective following inflammation-sensitized hypoxic-ischemic encephalopathy (8). This has also been described clinically in a small single-center study (9) and might be one of the reasons, why TH has failed to improve outcome in low- and middle-income countries (10). Early identification of asphyxiated newborns, who do not benefit from cooling remains one of the major challenges, as early biomarkers are lacking. Routinely used clinical blood biomarker identifying newborns with concomitant perinatal sepsis have not revealed robust results. C-reactive protein (CRP) response, being used as one of the broad markers identifying newborns with sepsis, is delayed by TH and non-specific for perinatal sepsis (11) as it may just reflect the inflammatory response to NE. Interleukin-6 (IL-6), being used as early and more specific blood biomarker to perinatal infection and early-onset sepsis in the clinical setting, has been shown to be correlated with CRP peak values at day 4 in cooled asphyxiated newborns, not reflecting early-onset sepsis (12). Serial IL-6 measurements have been suggested to help identifying newborns with inflammation-sensitized NE (13). However, conclusive data are lacking.

There is no obligatory national registry for cooled asphyxiated neonates in Germany. Hence, no national short- or long-term outcome data can be analyzed. As part of the German Hypothermia Network, a research collaboration of 85 hospitals, we previously performed an online survey on routine clinical practices of cooled asphyxiated newborns (14). The survey found a wide heterogeneity in treatment practices of cooled asphyxiated newborns. To present data on cooled asphyxiated newborns in Germany, we analyzed data from two large university neonatal intensive care units with similar guidelines for routine care of asphyxiated infants. The aim of this study was to evaluate if elevated biomarkers used routinely to diagnose early neonatal sepsis are associated with adverse short-term outcomes (death or abnormal MRI score).

## Methods

### Data Collection

Data from sixty-four term (*n* = 64, ≥ 37^+0^ weeks of gestation) or near-term (*n* = 3; 36^+0^ to 36^+6^ weeks of gestation) newborns born between 2009 and 2018 from two NICUs (highest level of care) in Germany were retrospectively analyzed with ethical permission (19-8556-BO, 18-8191-BO, 2018-270-ProspDEuA, 2018-270-1). Twenty-one newborns were born at (*n* = 12) or transferred to (*n* = 9) the first NICU (University Hospital Duesseldorf) and forty-six newborns were born at (*n* = 27) or transferred to (*n* = 19) the second NICU (University Hospital Essen). None of the newborns were cooled during transport. No cooled newborns were excluded.

All newborns were treated with whole body hypothermia for 72 h, starting within the first 6 h after birth, maintaining a rectal temperature of 33–34°C due to neonatal encephalopathy (NE). Similar treatment protocols were used in both NICUs. Newborns fulfilled the entry criteria for hypothermia therapy as used in the large randomized-controlled cooling trials [Apgar score ≤ 5 and/or ongoing resuscitation at 10 min, abnormal blood gases with a pH < 7.0 or base deficit ≥ 16 mmol/L as the immediate criteria for perinatal asphyxia, abnormal neurologic examination as the second criterion, and moderate or severe abnormalities on amplitude-integrated encephalography (aEEG) or seizures as the third]. Seizures were defined clinically and/or by aEEG.

Data for gestational age, gender, birth weight, birth place (inborn/outborn), Apgar scores at 5 and 10 min, lowest pH-, base excess- and lactate-within the first hour after birth, survival, degree of encephalopathy before initiation of cooling (Sarnat Score), onset and treatment of subclinical or clinical seizures before and during the cooling period and until the end of rewarming, time to normal trace using aEEG monitoring and time when cooling was initiated and when target temperature was reached were recorded for each newborn. Additionally, data regarding perinatal sepsis and infection was collected including maternal fever (≥38.5°C) within 24 h before birth, prolonged rupture of membranes (>18 h), maternal or neonatal positive blood culture results and maximum maternal CRP and leucocyte levels. In case of a positive neonatal blood culture, timing of positivity [early-onset sepsis (<72 h after birth, EOS) vs. late-onset sepsis (>72 h after birth, LOS)] and details of organisms were collected. Blood parameters obtained from the newborn included levels of interleukin (IL)-6 before initiation of cooling and CRP, leucocyte and platelet counts before initiation of cooling and thereafter within the first 24 h of cooling, day three of cooling (48–72 h of cooling) and at the end of rewarming. As the frequency of data sampling for CRP, leucocytes and platelets was based on clinical decision, the median was calculated for each 24-h time window, if needed. Due to clinical protocols, no CRP, leucocyte and platelet counts were available for the second day of cooling (24–48 h of cooling). We calculated area under the curve (AUC) for CRP, leucocytes and platelets using the trapezium rule, as previously described (6).

### Outcome Definition

As standardized long-term outcome (e.g., Bayley Scales of Infant Development) was not available in our cohort, short-term outcome was assessed using magnetic resonance imaging (MRI). Additionally, as aEEG is a good prognostic outcome predictor in cooled asphyxiated newborns (15), we used aEEG results in our study to further validate its predictivity.

MRI data was available in 60/67 newborns included in the study. MRI was performed on postnatal day 5–7 and MRI outcome was evaluated using a standardized scoring system, which defines MRI outcome depending on severity and location of brain injury on T1 and T2 weighted images and is correlated with long-term neurodevelopmental outcome in cooled asphyxiated newborns (16). In brief, basal ganglia and watershed scores were evaluated and the basal ganglia/watershed (BG/W) score was used as final outcome score. MRI scores were evaluated by three independent individuals based on the original MR images (T1 and T2 weighted images). Images were scored as following: 1 = no injury, 2 = mild injury, 3 = moderate injury, 4 = severe injury. MRI outcome was scored as good (BG/W score 0–2) or adverse (BG/W score > 2). All MRI scoring was performed by individuals blinded to the clinical information.

aEEG data was available in 65/67 newborns included in the study (MRI data was available in the two newborns with missing aEEG data). Single-use needle electrodes were used to record the biparietal EEG signal (locations equivalent to C3-P3 and C4-P4 of a standard EEG). Continuous recording was started from admission soon after birth until the end of the rewarming phase (Brainz or Olympic Brainz Monitor, Natus, USA). The aEEG background voltage changes were continuously recorded, and voltage criteria were used to measure the voltage level of the upper and lower margin of the time-compressed aEEG traces (17). We differentiated between the physiological continuous normal voltage and discontinuous normal voltage patterns and burst suppression, continuous low voltage and flat trace as pathological patterns. aEEG was scored as normal, when time to normal aEEG trace was achieved within 48 h after initiation of cooling (15). Seizures on aEEG were scored as pathological. aEEG recordings were analyzed by three individual investigators who were blinded to the clinical information.

MRI outcome was used as the primary outcome measure, defining favorable or adverse outcome in our study. Adverse outcome was defined as death or adverse MRI findings, defined as BG/W score > 2. From the nine newborns who died, MRI data were available in three. aEEG data were available for all nine newborns. We additionally correlated aEEG to MRI outcome in our study, aiming to support the use of aEEG as outcome predictor in cooled asphyxiated newborns and to strengthen our results.

### Data Analysis

Statistical analysis was performed using SPSS 26 (SPSS, Chicago, Ill., USA). Non-parametric data was analyzed using the Mann-Whitney U test for analysis between groups. Multivariate analysis using stepwise binary logistic regression was performed with MRI outcome as the dependent variable. Independent variables were APGAR scores at 5 and 10 min, first pH, seizures (yes/no), anticonvulsant treatment, aEEG time to normal trace, IL-6 before initiation of cooling, CRP 48–72 h after birth, CRP after cooling, leucocytes after cooling, platelets 48–72 h after birth, platelets after cooling. MRI and aEEG were correlated using Pearson correlation. To assess the predictive value of IL-6 before initiation of cooling, CRP 48–72 h after birth and CRP after cooling, receiver operating characteristic (ROC) curves were used. The AUC was calculated based on the ROC curves. A *p*-value of ≤0.05 was considered as significant. Descriptive data are presented as mean ± standard deviation (SD).

## Results

There were 31 male and 36 female newborns included in the study. Mean ± SD gestational age and birth weight were 39^+1^ ± 1^+3^ weeks and 3,231 ± 693 grams, respectively, and there was no significant difference in distribution to the outcome groups. Forty-nine newborns had favorable short-term MRI outcome, 18 had adverse short-term MRI outcome. All analyzed descriptive data are presented in Table 1. We found that APGAR scores at 5 and 10 min and pH values within the first hour after birth were significantly lower in newborns with adverse short-term MRI outcome (*p* < 0.05). There was no significant difference between the groups regarding level of encephalopathy (Thompson Score) prior to TH. Grade of encephalopathy (Sarnat score) prior to TH showed more severe scores in the adverse short-term MRI outcome group. Nine infants died within the first week after birth. No death occurred within the first 72 h after birth. Seizure rates were significantly higher in newborns with adverse short-term MRI outcome (78 vs. 39%, *p* = 0.002) resulting in a significantly higher rate of anticonvulsant treatment in newborns with adverse short-term MRI outcome (67 vs. 35%, *p* = 0.023). All data referring to perinatal sepsis and infection (maternal fever within 24 h before birth, prolonged rupture of membranes, maternal or neonatal positive blood culture results and maximum maternal CRP and leucocyte levels) did not differ significantly between groups (Table 1). The number of positive blood cultures was 31 vs. 56% in the good vs. adverse short-term MRI outcome group. The individual bacterial organisms are presented in Table 2. Ninety-two percent of positive blood cultures were found in cultures taken within the first 72 h after birth (EOS).

**Table 1:** Descriptive data of the analyzed cohort.

	**Good outcome (*n* = 49)**	**Adverse outcome (*n* = 18)**	***p*-value**
Birth weight (mean ± SD kg)	3,225 (±700)	3,251 (±697)	0.889
Gender (*n*, % male)	23 (47)	8 (44)	0.859
Gestational age (mean ± SD week)	38 (±3)	39(±2)	0.133
APGAR score 5 min [median (range)]	5 (0–10)	3 (0–6)	** <0.001**
APGAR score 10 min [median (range)]	7 (1–10)	4 (0–7)	** <0.001**
First pH (mean ± SD)	6.88 (±0.16)	6.76 (±0.20)	**0.020**
First base excess (mean ± SD)	20.5 (±6.7)	22.4 (±7.5)	0.329
First lactate level (mean ± SD)	11.9 (±5.7)	14.4 (±6.4)	0.150
Thompson score before cooling (mean ± SD)	10 (±5.5)	11 (±5.3)	0.328
HIE Grade before cooling (*n* = mild, *n* = moderate, *n* = severe)	16 = mild, 23 = moderate, 8 = severe	6 = moderate, 12 = severe	
Inborn (*n*, %)	29 (60)	11 (61)	0.889
Death	0	*n* = 9 (50%)	**0.001**
Seizures (*n*, %)	19 (39)	14 (78)	**0.002**
Anticonvulsant treatment (*n*, %)	17 (35)	12 (67)	**0.023**
Positive blood culture (*n*, %)	15 (31)	10 (56)	0.054
Maternal fever (*n*, %)	5 (10)	2 (11)	0.158
Prolonged rupture of membranes (*n*, %)	8 (16)	4 (22)	0.663
Maximum maternal CRP (mean ± SD)	5.6 (±7.3)	9.4 (±9.0)	0.194
Maximum maternal leukocytes (mean ± SD)	15.8 (±7.6)	12.7 (±3.8)	0.137
Maternal positive blood culture (*n*)	4	2	0.698
Time to target temperature (minutes, mean ± SD)	166 (±100)	180 (±117)	0.620
aEEG time to normal trace (hours, mean ± SD)	11 (±13.7)	150 (±140)	**0.001**
Time point MRI (days, mean ± SD)	8 (±6)	6 (±2)	0.250
MRI outcome (Basal ganglia/watershed score)	34 = no injury (1), 14 = mild injury (2), *n* = 1 no MRI	7 = moderate injury (3), 5 = severe injury (4), *n* = 6 no MRI	
IL-6 before initiation of cooling (ng/l, mean ± SD)	101.8 (±1,12.4)	1,707 (±2,053.2)	**0.008**
CRP before initiation of cooling (mg/dl, mean ± SD)	0.6 (±1)	0.9 (±1.4)	0.356
CRP first 24 h of cooling (mg/dl. mean ± SD)	1.0 (±1.8)	1.4 (±2.1)	0.495
CRP 48–72 h of cooling (mg/dl, mean ± SD)	2.1 (±2.6)	4.9 (±4.6)	**0.031**
CRP after cooling (mg/dl, mean ± SD)	1.5 (±2.2)	4.8 (±4.4)	**0.008**
AUC CRP (mg/dl, mean ± SD)	1.4 (±1.7)	3.0 (±2.6)	**0.025**
Leucocytes before initiation of cooling (×10^9^/l, mean ± SD)	20.5 (±6.6)	22.3 (±7.2)	0.398
Leucocytes first 24 h of cooling (×10^9^/l, mean ± SD)	16.0 (±6.6)	17.1 (±7.2)	0.590
Leucocytes 48–72 h of cooling (×10^9^/l, mean ± SD)	11.4 (±5.7)	14.0 (±6.7)	0.184
Leucocytes after cooling (×10^9^/l, mean ± SD)	11.0 (±4.7)	16.4 (±8.5)	**0.036**
AUC Leucocytes (×10^9^/l, mean ± SD)	14.4 (±4.6)	17.3 (±5.7)	0.069
Platelets before initiation of cooling (×10^3^/μl, mean ± SD)	206 (±77)	188 (±79)	0.422
Platelets first 24 h of cooling (×10^3^/μl, mean ± SD)	173 (±55)	166 (±55)	0.642
Platelets 48–72 h of cooling (×10^3^/μl, mean ±SD)	143 (±52)	180 (± 61)	**0.033**
Platelets after cooling (×10^3^/μl, mean ± SD)	133 (±60)	179 (±49)	**0.004**
AUC Platelets (×10^3^/μl, mean ± SD)	163 (±52)	176 (±41)	0.250

*Bold values indicate statistical significance*.

**Table 2:** Bacterial organisms detected via positive blood culture.

	**Good outcome (*n* = 15)**	**Adverse outcome (*n* = 10)**
Early-onset sepsis	*E. coli* (*n* = 5) Group B streptococcus (*n* = 5) *Staphylococcus aureus* (*n* = 2) Klebsiella spp. (*n* = l) Enterobacter spp. (*n* = l)	Enterobacter spp. (*n* = 2) Enterococci spp. (*n* = 2) Group B streptococcus (*n* = 2) Group A streptococcus (*n* = l) Pseudomonas spp. (*n* = l) *Staphylococcus aureus* (*n* = l)
Late-onset sepsis	Serratia spp. (*n* = l)	Coagulase-negative staphylococcus (*n* = l)

*EOS, early-onset sepsis; LOS, late-onset sepsis*.

**Figure 1:**
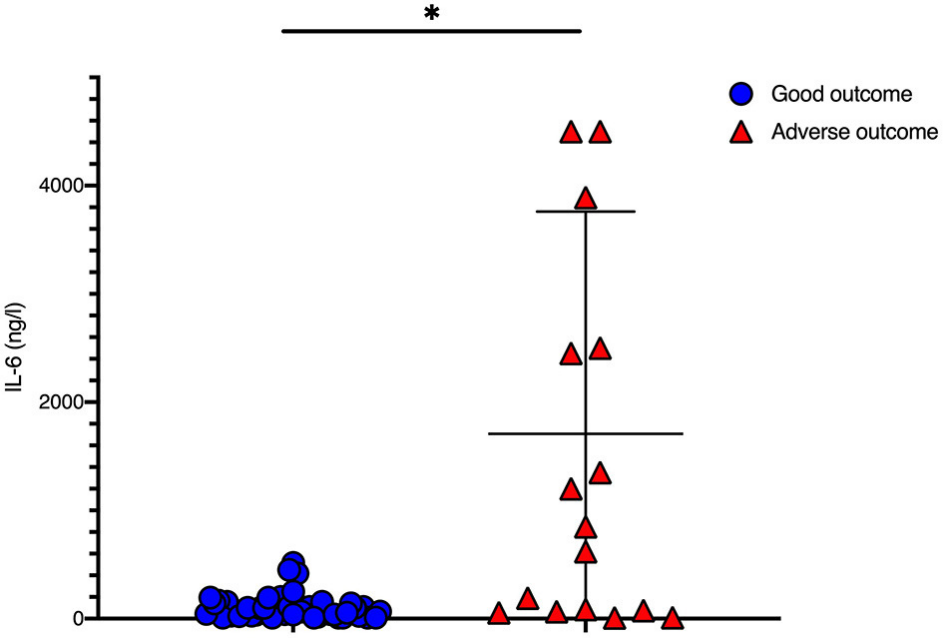
IL-6 values before initiation of therapeutic hypothermia: IL-6 values were significantly higher in newborns with death or adverse MRI findings, defined as BG/W score > 2. Data presented as mean ± standard deviation (SD), *p < 0.05.

**Figure 2:**
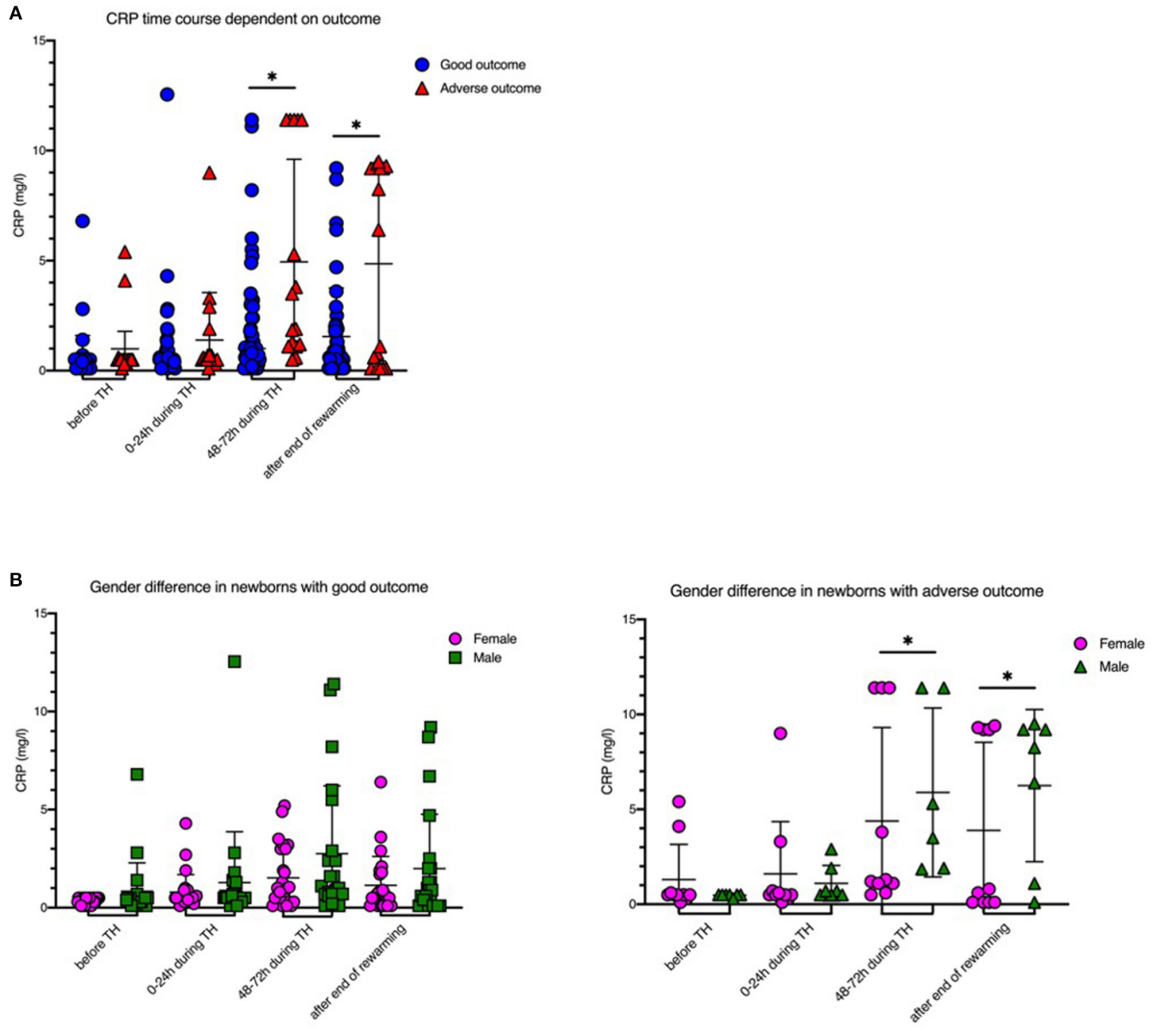
CRP levels before, during and after therapeutic hypothermia. **(A)** CRP levels were higher in newborns with death or adverse MRI findings, defined as BG/W score > 2. **(B)** Males had increased CRP values compared to females. Males with death or adverse MRI findings had significantly increased CRP values at the end of the cooling phase and after rewarming. Data presented as mean ± standard deviation (SD), *p < 0.05.

**Figure 3:**
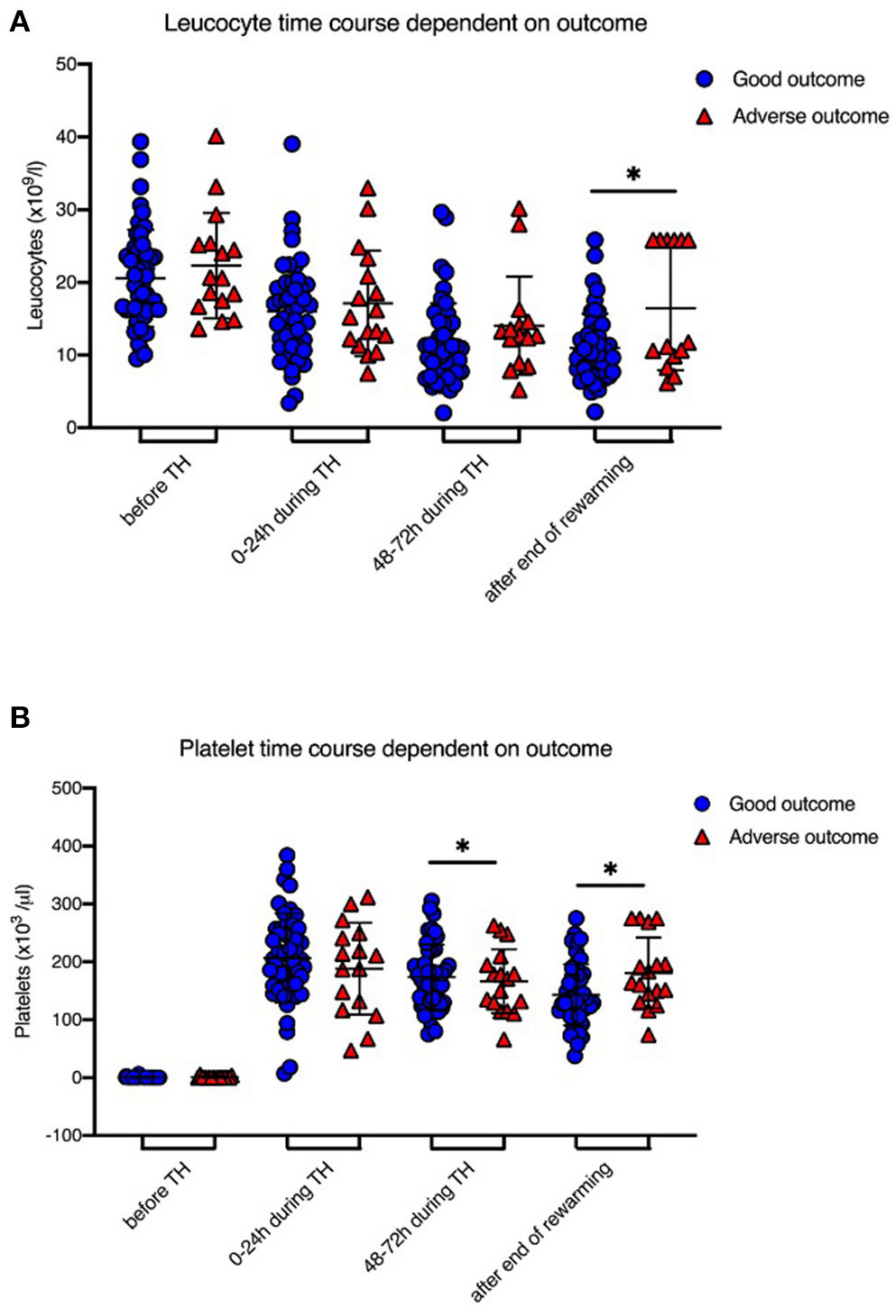
Leucocyte and Platelet levels during therapeutic hypothermia. **(A)** Leucocyte levels were decreased after initiation of TH in both groups, however leucocytes were significantly higher in newborns with death or adverse MRI findings, defined as BG/W score > 2, after rewarming. **(B)** Platelet counts were significantly higher at the end of TH and after rewarming in newborns with death or adverse MRI findings. Data presented as mean ± standard deviation (SD), *p < 0.05.

IL-6 levels before initiation of TH were significantly higher in newborns with adverse short-term MRI outcome [1,707 (±2,053.2) ng/l vs. 101.8 (±112.4) ng/l, *p* = 0.008; Table 1, Figure 1]. CRP time course during TH is shown in Figure 2. We found that CRP levels were higher at all time points in newborns with adverse short-term MRI outcome, being significantly higher at the end of the cooling phase [2.1 (±2.6) vs. 4.9 (±4.6) mg/dl, *p* = 0.031] and after rewarming [1.5 (±2.2) vs. 4.8 (±4.4) mg/dl, *p* = 0.008] (Figure 2A). Additionally, AUC CRP values were significantly higher in newborns with adverse short-term MRI outcome [1.4 (±1.7) vs. 3.0 (±2.6) mg/dl, *p* = 0.025; Table 1]. We observed a gender specific difference in CRP response and outcome correlation in our study. As shown in Figure 2B, males with adverse short-term MRI outcome had significantly increased CRP values at the end of the cooling phase and after rewarming compared to females with adverse short-term MRI outcome [3.8 (±2.6) vs. 6.2 (± 4.0) mg/dl, *p* < 0.05]. Leucocyte levels were not significantly different between the groups before initiation of TH (Figure 3A). We found a decrease of leucocyte levels after initiation of TH in both groups; however, leucocytes were significantly higher in newborns with adverse short-term MRI outcome after rewarming [16.4 (±8.5) × 10^9^/l vs. 11.0 (±4.7) × 10^9^/l, *p* = 0.036]. Similar results were found for platelet counts with a decrease in platelet numbers after initiation of TH and significantly higher platelet counts at the end of TH and after rewarming in newborns with adverse short-term MRI outcome (*p* = 0.033 and 0.004, respectively) (Figure 3B). There was no gender specific difference in leucocyte or platelet counts between the outcome groups.

**Figure 4:**
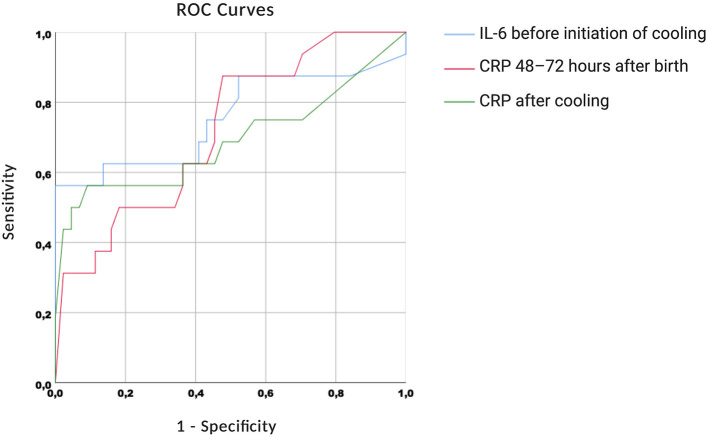
Receiver operating characteristic curves.

The ROC curves are shown in Figure 4. We found high AUC measures for IL-6 before initiation of cooling [0.755 (0.586–0.924)], CRP 48–72 h after birth [0.719 (0.577–0.862)] and CRP after cooling [0.690 (0.508–0.873)].

### Correlation of Clinical Data With Short-Term MRI Outcome

We found that aEEG time to normal trace was significantly longer in newborns with adverse short-term MRI outcome [180 (±117) h vs. 11 (±9.7) h, *p* = 0.001]. Using binary logistic regression, we found that aEEG time to normal trace was the strongest independent outcome predictor in our study (Table 3). When we analyzed the data without using aEEG time to normal trace in the equation, we found that CRP levels after rewarming were significantly associated with adverse short-term MRI outcome (*p* = 0.016). All other independent variables analyzed in the regression were not correlated with adverse short-term MRI outcome (all variables used were the ones that differed significantly between outcome groups in the descriptive analysis).

**Table 3:** Results of multivariate analysis using binary logistic regression.

**Independent variable**	**Coefficient**	***p*-value**	**Lower 95% CI**	**Upper 95% CI**
aEEG time to normal trace, hours	0.067	0.026	1.008	1.135
**Without aEEG time to normal trace**				
CRP after cooling	0.444	0.016	1.086	2.238

*Independent variables: APGAR 5, APGAR 10, first pH, seizures (yes/no), anticonvulsant treatment, aEEG time to normal trace, IL-6 before initiation of cooling, CRP 48–72 h, CRP after cooling, leucocytes after cooling, platelets 48–72 h, platelets after cooling*.

As aEEG time to normal trace has been shown to be a significant outcome predictor in previous studies, we also found a highly significant correlation between aEEG and short-term MRI outcome in our study (*p* < 0.001, *r*^2^ = 0.242).

## Discussion

The current study investigated a possible correlation of routinely used blood biomarkers for perinatal infection and sepsis with adverse short-term MRI outcome in cooled asphyxiated infants. We found a significant correlation between elevated serum levels of the cytokine IL-6 before initiation of therapeutic hypothermia and adverse short-term MRI outcome. During therapeutic hypothermia newborns with adverse short-term MRI outcome also had increased CRP levels compared to newborns with good outcome, being significantly higher after 48 h of cooling treatment and after the end of therapeutic hypothermia. Interestingly, we observed a gender-specific difference in CRP response and outcome correlation, as males with adverse short-term MRI outcome had significantly increased CRP values at the end of the cooling phase and after rewarming.

Neonatal sepsis remains an important risk-factor for neonatal mortality and is suspected to alter the neuroprotective effect of therapeutic hypothermia in term, or near-term asphyxiated newborns (8, 10, 18). Clinical manifestation of neonatal sepsis is often non-specific and overlaps with symptoms of non-infectious inflammation of different origin, such as respiratory distress syndrome or neonatal hypoglycemia. One of the gold standards, diagnosing neonatal sepsis, is a positive blood culture. However, it is well-described, that specific detection of bacteria via positive blood culture is low in neonates (19). In our cohort of cooled asphyxiated newborns, we did not find significant differences in blood culture positivity in the different outcome groups. Of notice, we found a much higher incidence of positive blood cultures in our cohort, compared to the previous cohorts of asphyxiated newborns. Most (92%) of the positive blood cultures in our cohort were found in cultures obtained within the first 72 h after birth (EOS). The detection of maternal positive blood cultures was much lower, compared to the neonatal cultures. The high rate of presumed infection-sensitized encephalopathies might have influenced the outcome in our cohort, as we found in pre-clinical studies that therapeutic hypothermia is not beneficial following inflammation-sensitized hypoxic-ischemic brain injury (8). Though, we did not observe a major difference in death or unfavorable short-term MRI outcome in our cohort compared to the historical cohorts described in the literature (1).

Previous studies investigating the diagnostic markers of neonatal sepsis revealed a variety of possible parameters, such as procalcitonin, CRP, different cytokines (e.g., IL-6, IL-10) and micro-RNAs (e.g., miR-155), but none of them show ideal characteristics in sensitivity and specificity (20, 21). Sharma et al. found that CRP is an easily available but non-specific marker for neonatal sepsis as there are different reasons for increased CRP levels such as premature rupture of membranes, meconium aspiration syndrome, or maternal fever during labor. Nevertheless, a meta-analysis showed variable sensitivity (30–80%) but good specificity (83–100%) for diagnosis of neonatal sepsis using CRP levels within the first 28 days of life (22). In asphyxiated newborns, CRP levels are increased within the first 7 days and the increase corresponds to severity of brain injury (23). This increase has been shown to be delayed by therapeutic hypothermia (11, 24). However, it remains unclear whether neonatal sepsis is exclusively responsible for the rise of CRP in cooled asphyxiated newborns. Hypoxia-ischemia clearly induces inflammation (25), also raising pro-inflammatory markers, such as CRP or IL-6 (26). Additionally, severity of hypoxia-ischemia influences the peak levels of CRP values (27). In our study, we also observed an increase of CRP values over time with a significant increase in the newborns with adverse short-term MRI outcome. We also found a difference dependent on gender. Al Mamun et al. have shown that inflammatory responses are sex-specific following experimental hypoxic-ischemic encephalopathy (28). They found that male mice had worse HIE outcomes than females. This has also been shown by other research groups and might contribute to sex-specific neuroprotection following experimental brain injury (29). In our study peak CRP levels were reached 48 h after birth. Newborns with adverse short-term MRI outcome presented with consisting high CRP levels thereafter, whereas newborns with good short-term MRI outcome showed a decrease in CRP levels after its peak.

IL-6 has been investigated to be an appropriate and early marker for neonatal sepsis having 90% sensitivity but lower negative predictive value of 67.4% when measured within the first hours of neonatal sepsis (30). IL-6 is produced in the early phase of the inflammatory response. As previously shown IL-6 levels are hardly detectable in healthy humans, but are highly expressed during inflammation (31). Following perinatal asphyxia, IL-6 levels in the cerebrospinal fluid have been shown to correlate with severity of brain injury (32). Chalak et al. showed that high IL-6 levels within the first 24 h after birth correlate with abnormal neurological outcome in cooled asphyxiated newborns (33). The new finding of our study is the correlation of high IL-6 serum levels within the first 6 h after birth and adverse short-term MRI outcome. This may be considered as the first response of inflammation, which might alter the neuroprotective effect of therapeutic hypothermia in cooled asphyxiated newborns after inflammation-sensitization. There is experimental evidence that the neuroprotective effect of TH is lacking when combined with inflammation (8, 18). Osredkar et al. found in preclinical studies, that TH is not neuroprotective following inflammation-sensitization in newborn rats. However, no gender difference was observed in these preclinical studies.

Due to a lack of long-term outcome data, we assessed short-term outcome using MRI and aEEG. Barkovich et al. first described a MRI scoring system aiming to predict long-term neurodevelopmental outcome in newborns with perinatal asphyxia (16). They developed a scoring system based on injuries in the basal ganglia (BG) and watershed (W) regions of the newborn brain and found a strong association especially of the BG/W score with neuromotor outcome at 3 and 12 months and cognitive outcome at 12 months (16). In cooled asphyxiated newborns, the Barkovich MRI scorning system has shown to be still a reliable predictor of adverse outcome at 2 years (34) and therefore it was used in our study. Additionally, we used aEEG to validate and strengthen the MRI scoring results. aEEG is a reliable outcome predictor for long-term neurodevelopmental impairment in cooled asphyxiated newborns as confirmed in previous studies (15). Persistently abnormal aEEG pattern >48 h after birth is associated with adverse outcome in cooled asphyxiated newborns, with a positive predictive value of 85% (35). In our study, we confirmed a very strong correlation between aEEG time to normal trace and MRI outcome.

There are limitations to our study. First, there is the lack of a standardized neurodevelopmental long-term outcome assessment. As MRI and aEEG have been described to correlate with long-term outcome in cooled asphyxiated newborns, we believe that our findings represent long-term outcome. However, long-term neurodevelopmental outcome tests remain the gold standard and cannot be replaced with short-term assessments. In general, the Bayley Scales of Infant development are used as the standardized neurodevelopmental outcome test with 20–28 months of life in cooled asphyxiated newborns (1). When we analyzed our data, we found that most of the infants and families did not attend the invitation to follow-up at 20–28 months. A second limitation of this study is the retrospective design. This study only provides basic data which has been collected during the clinical routine and based on clinical guidelines. As it was not the aim of our study and due to the relatively small sample size, we did not estimate or calculate cut-off values for the analyzed blood biomarkers with short-term MRI outcome. Nevertheless, it is useful to get an insight into the current medical treatment of cooled asphyxiated newborns in two large German NICUs. We have previously shown that there is a large variability in treatment practices of cooled asphyxiated newborns in Germany (14). Therefore, a national data registry is urgently needed. Last, we found a high incidence of positive blood cultures in our study. Therefore, the inflammation in our cohort might be additionally triggered by neonatal infection. We did not observe a significant difference of positive blood cultures between our outcome groups (*p* = 0.054) and of interest, the number of positive blood cultures was higher in the group with good short-term MRI outcome compared to the group with adverse short-term MRI outcome. Therefore, we cannot distinguish whether infection induced inflammation was a contributing factor, leading to differences between the assessed blood biomarkers in our retrospective study.

In summary we found that routinely used blood biomarkers may be helpful early identifying newborns that are at high risk for an unfavorable outcome and in need of close neurodevelopmental follow-up. Since IL-6 and CRP are no absolute markers for the diagnosis of inflammation in terms of neonatal sepsis and might have been influenced by a large proportion of positive blood cultures in our cohort, the conclusion of our data has to be confirmed by other cooling centers. Recognizing which depressed neonates have infections that might further induce inflammation may enable future cooling therapies and further prospectively assign asphyxiated neonates to interventions in multicenter randomized controlled studies.

## Data Availability Statement

The original contributions presented in the study are included in the article/supplementary material, further inquiries can be directed to the corresponding author/s.

## Ethics Statement

The studies involving human participants were reviewed and approved by Ethics committee University Hospital Essen Ethics committee University Hospital Duesseldorf. Written informed consent from the participants' legal guardian/next of kin was not required to participate in this study in accordance with the national legislation and the institutional requirements.

## Author Contributions

MR and HS have planned and designed the study. MR, SG, MM, and AS have collected the data. MR, SG, MM, AS, and HS have analyzed the data. MR, AS, TH, UF-M, and HS have written and corrected the manuscript. All authors contributed to the article and approved the submitted version.

## Conflict of Interest

The authors declare that the research was conducted in the absence of any commercial or financial relationships that could be construed as a potential conflict of interest.
